# Targeting Metabolic Reprogramming to Improve Breast Cancer Treatment: An In Vitro Evaluation of Selected Metabolic Inhibitors Using a Metabolomic Approach

**DOI:** 10.3390/metabo11080556

**Published:** 2021-08-22

**Authors:** Anaïs Draguet, Vanessa Tagliatti, Jean-Marie Colet

**Affiliations:** Laboratory of Human Biology and Toxicology, Faculty of Medicine and Pharmacy, University of Mons, 7000 Mons, Belgium; anais.draguet@umons.ac.be (A.D.); vanessa.tagliatti@umons.ac.be (V.T.)

**Keywords:** ^1^H-NMR, metabonomic, breast cancer, metabolic inhibitors, Doxorubicin, Oxamate, CB-839, D609

## Abstract

Characteristic metabolic adaptations are recognized as a cancer hallmark. Breast cancer, like other cancer types, displays cellular respiratory switches—in particular, the Warburg effect—and important fluctuations in the glutamine and choline metabolisms. This cancer remains a world health issue mainly due to the side effects associated with chemotherapy, which force a reduction in the administered dose or even a complete discontinuation of the treatment. For example, Doxorubicin is efficient to treat breast cancer but unfortunately induces severe cardiotoxicity. In the present in vitro study, selected metabolic inhibitors were evaluated alone or in combination as potential treatments against breast cancer. In addition, the same inhibitors were used to possibly potentiate the effects of Doxorubicin. As a result, the combination of CB-839 (glutaminase inhibitor) and Oxamate (lactate dehydrogenase inhibitor) and the combination of CB-839/Oxamate/D609 (a phosphatidylcholine-specific phospholipase C inhibitor) caused significant cell mortality in both MDA-MB-231 and MCF-7, two breast cancer cell lines. Furthermore, all inhibitors were able to improve the efficacy of Doxorubicin on the same cell lines. Those findings are quite encouraging with respect to the clinical goal of reducing the exposure of patients to Doxorubicin and, subsequently, the severity of the associated cardiotoxicity, while keeping the same treatment efficacy.

## 1. Introduction

Cancer is reported as a major public health issue worldwide. According to the World Health Organization (WHO), 18.1 × 10^6^ new cancers were diagnosed in 2018, and 9.6 × 10^6^ patients died of their disease. These statistics could reach 26.4 × 10^6^ new cancer cases and 17 × 10^6^ related deaths per year in 2030. The main reasons behind this sad projected evolution are the increasing and aging world population, as well as the poor quality of life in some regions. The most frequently reported cancers are breast, prostate, lung and colon cancers [1].

Defined in 2000 [2] and updated in 2011 [3], the cancer hallmarks help to better understand this pathology and to optimize the development of new treatments. A hallmark is a characteristic feature acquired by cancer cells as compared to healthy cells [4]. Therefore, Hanahan and Weinberg defined six of these hallmarks: cell death resistance, angiogenesis induction, immortality, invasion and metastases activation, growth suppressor signal escapement and proliferative signaling autonomy [2]. More recently, this list was updated with the addition of four new hallmarks: cellular energetics deregulation, immune destruction escapement, genomic instability and inflammation, which promote tumor development [3].

In the present study, we focused on cell energetic deregulation, also known as metabolic reprogramming. Indeed, for a few years now, some cancer metabolic hallmarks have gained attention: the deregulation in glucose and amino acid cellular uptakes, the cellular consumption of opportunistic nutrients, the deviated use of glycolysis and Krebs cycle intermediates for macromolecule biosynthesis and nicotinamide dinucleotide phosphate (NADPH) production, a higher nitrogen demand, the metabolite driven alteration of gene regulation and metabolic interactions with the microenvironment [5]. Some metabolic pathways are known to be disrupted in cancer, among which the Warburg effect, glutaminolysis and choline metabolism are mostly studied.

Glucose metabolism is disrupted in cancer cells. Glucose is consumed in larger quantities to sustain higher lactate production, a phenomenon that is also possible in noncancer cells but only in hypoxic conditions. Such metabolic adaptations in cancer cells mainly support the increased need of ATP [6], the release of lactate in the extracellular fluid to ensure tissue environment acidification and promote tumorigenesis, escape from the immune system, intracellular acidification and metabolic pathway inhibition avoidance and, finally, metabolic coupling between cells within the tumor [6,7,8].

Glutamine is an amino acid essential for many fundamental functions in cells: redox balance, lipid and protein anabolism, the nitrogen rate, amino acid transport with, for example, an antiport system for leucine transport, glutathione synthesis and anaplerosis, namely Krebs cycle supply [5,6,9,10]. In cancer cells, glutamine is involved in cellular survival and growth. From a metabolic point of view, glutamine is transformed into glutamate in a reaction catalyzed by glutaminase (GLS) [9].

Finally, the choline content is also boosted in cancer cells following a disruption of its metabolism [11]. This compound is essential for membrane maintenance and synthesis. It is also a methyl donor [12]. In choline metabolism, some important compounds are produced, such as, for example, phosphatidylcholine (PtdChp), a membrane component, and phosphocholine (PCho), a second messenger in cells. The boost in choline metabolism leads to higher PCho production, total Choline increase (tCho) and a switch between PCho and glycerophosphocholine (GPCho), where PCho becomes the most abundant choline compound instead of GPCho [11,13].

The above-mentioned metabolic adaptations (glucose, glutamine and choline) can be related to a common denominator: HIF-1 (Hypoxia Inducible Factor) [9,13,14,15], a transcription factor composed of two subunits: HIF-1α and HIF-1β. Both subunits are constitutively expressed, but only the regulation of HIF-1α expression is dependent on the oxygen supply and, consequently, very sensitive to hypoxia. Indeed, contrary to its synthesis, the degradation of HIF-1α is regulated by oxygen-dependent mechanisms in addition to transcription factors and oncogenes [16,17,18]. The HIF-1α protein expression is absent in healthy tissue but raises up to 53% of cancers [14]. The expression of this transcription factor is associated with tumor aggressivity and treatment resistance in some cases [16]. In patients presenting a cancer type with a high level of HIF-1α, a higher mortality rate is observed.

In this study, we focused on metabolic adaptations in breast cancer that cover 16% of cancer in women worldwide [19]. The risk factors are essentially the patient’s age, genetic predisposition, the exposure to ionizing radiations and a lack of physical activity [20]. Breast cancer can be classified according to different methods, like, for example, histological or molecular classifications. The latter depends on surface markers: estrogen receptor (ER), progesterone receptor (PR) and human epidermal growth factor receptor 2 (HER2). These receptors define four phenotypes: luminal A and B cancers, which are positive for ER and PR and negative for HER2, basal-like cancer, which is positive for HER2 and negative for ER and PR and, finally, triple-negative cancers, negative for the three receptors [21].

Despite the medical progress in the field, breast cancer is still associated with a high mortality rate mainly due to the side effects of the current treatments, which force a reduction in the administered dose or even a complete discontinuation of the treatment.

A frequent treatment in breast cancer is Doxorubicin, an antibiotic molecule extracted from *Streptomyces peucetius*. This molecule has been used in chemotherapy since the sixties [22]. Doxorubicin uses two active mechanisms: DNA intercalation and free radical generation [23]. Although very efficacious, some side effects are unfortunately recognized in Doxorubicin treatment, especially severe acute and chronic cardiotoxicity. Acute cardiac toxicity is linked to reversible myopericarditis, left ventricular dysfunction and arrythmia. These effects occur on the day of Doxorubicin administration. The chronic effects are consequences of congestive cardiac insufficiency appearing in the months following exposure to the drug [22]. The side effects originate from off-target mechanisms. Two hypotheses are currently being debated: the formation of a metabolite (Doxorubicinol) with free radical production and mitochondria disruption with the reactive species of oxygen (ROS) inducing oxidative stress [23,24]. To avoid adverse effects, it is recommended to reduce the therapeutic dose at the risk of a lower efficacy. Additionally, to compensate and improve the treatment efficacy at low doses, it was proposed to combine Doxorubicin with metabolic inhibitors [25,26,27]. This was suggested by Warburg’s discovery on the importance metabolic switch in cancer cells. First, Doxorubicin was combined with only one single metabolic inhibitor at a time; however, with poor improvement efficacy, this led us to using an untargeted metabonomic approach to identify multiple metabolic pathways that were characteristic of the cancer types [28]. This strategy helped us in the selection of specific combinations of metabolic inhibitors. In the present study, we focused on breast cancer, for which the glutamine, glucose/lactate, choline and HIF-1 pathways were identified as the most crucial pathways for cell survival. Therefore, we decided to evaluate the impact of exposing breast cancer cell lines simultaneously to multiple metabolic inhibitors.

As in many other cancer types, breast cancer undergoes metabolic adaptations to sustain cell proliferation and survival. The aim of this study is to evaluate selected metabolic inhibitors on two breast cancer cell lines (the MDA-MB-231 cell line derived from triple-negative cancer and the MCF-7 cell line originating from estrogen and progesterone receptor cancer) in order to determine their efficacy as a potential therapy in breast cancer. In addition, their ability to potentiate the Doxorubicin effect was also evaluated with the clinical perspective of reducing the administered Doxorubicin dose and, consequently, the related cardiotoxicity in patients.

Besides viability and proliferation assays, an ^1^H-NMR-based metabolomic approach was mainly used in this study to assess the cellular metabolic variations in response to the tested metabolic inhibitors.

## 2. Results

### 2.1. Metabolic Signature of Breast Cancer Tested Cell Lines

**Figure 1:**
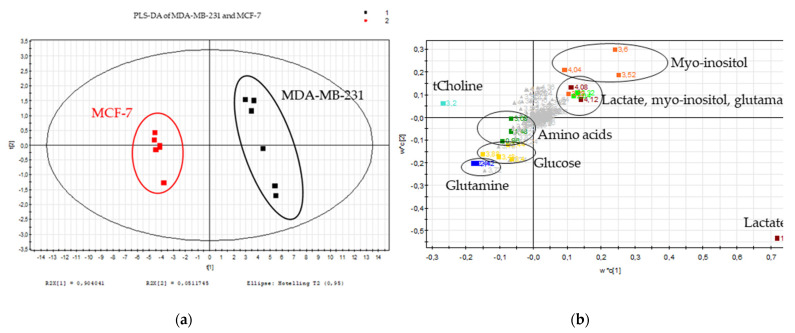
Metabolic signature of the breast cancer cell lines. (**a**) Scores plot and (**b**) loadings plot of the PLS-DA of the MDA-MB-231 (black) and MCF-7 (red) cellular polar extracts (*n* = 6); burgundy: lactate, orange: myo-inositol, light green: glutamate, dark green: amino acids (alanine and isoleucine), light blue: choline, dark blue: glutamine and yellow: glucose for 231 variables.

The metabolome of each selected breast cancer cell line was determined by ^1^H-NMR spectroscopy. A comparison between the metabolomes of both cell lines was carried out based on the multivariate data analysis of the spectral binned data. The PLS-DA score plots show a significant separation of both cell types (*p*-value = 5.25 × 10^−8^) (Figure 1). The loadings plot reveals that tCho, glucose, glutamine and the amino acids are more concentrated in MCF-7 cells, while lactate, myo-inositol and glutamate are more present in MDA-MB-231 cells (Figure 1). The metabolic pathways mostly modified in both cell lines are involved in the glucose, alanine, lactate, glutamine and choline pathway.

### 2.2. Assessment of the Efficacy of the Selected Metabolic Inhibitors

**Figure 2:**
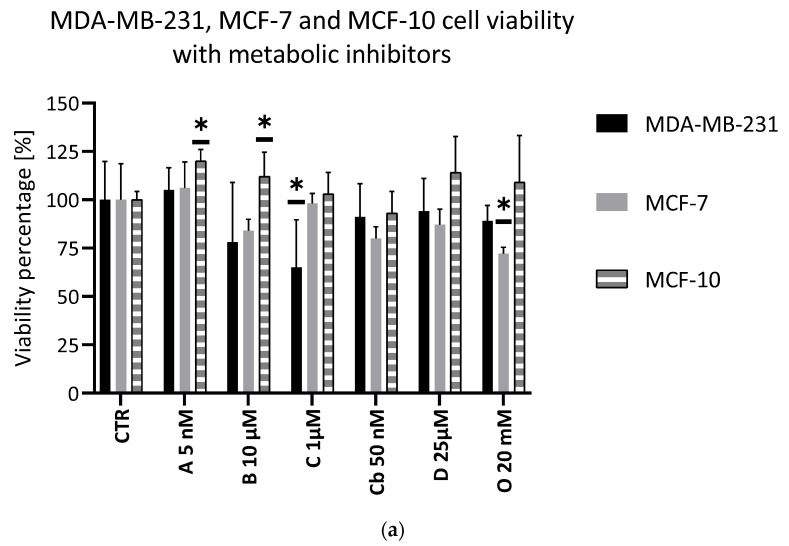

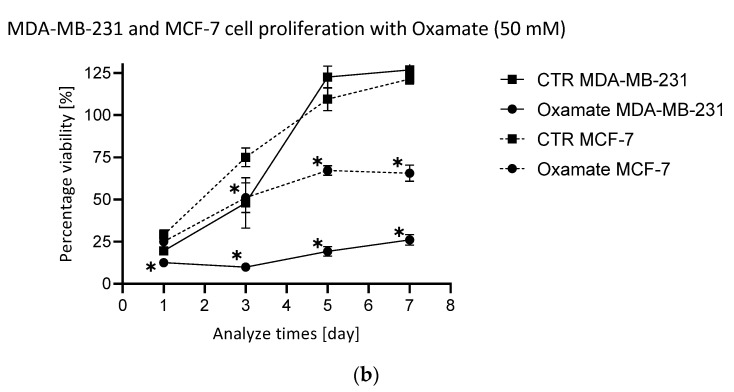
Metabolic inhibitor evaluation. (**a**) Viability test (crystal violet staining) after 72 h. Black: MDA-MB-231, Grey: MCF-7 and Hatched: MCF-10; CTR: control condition. A: AZD3965, B: 3-bromopyruvate, C: Chrysin, Cb: CB-839, D: D609 and O: Oxamate; *n* = 6, average ± SD, significance: * *p* < 0.05. (**b**) Proliferation test (crystal violet staining) during 7 days of measuring at days 1, 3, 5 and 7 of the cells treated with Oxamate 50 mM. Full lines: MDA-MB-231 and Hatched lines: MCF-7; *n* = 6, average ± SD, significance: * *p* < 0.05.

The efficacy of the metabolic inhibitors was evaluated in viability and proliferation assays on both cell lines. They showed a minimal impact on the cellular viability. In the MDA-MB-231 cell line, Chrysine showed a significant effect on the cell viability, while, in the MCF-7 cell line, Oxamate had the highest impact on the cell viability. Other inhibitors did not show any significance on the cell viability (Figure 2). Regarding the proliferation assay, only Oxamate induced a significant decrease of MDA-MB-231 cell proliferation (Figure 2).

The metabolic inhibitors showed a low impact on the MCF-10 cell line and noncancer breast cell line. No inhibitor could significantly decrease the viability. Interestingly, AZD3965 and 3-bromopyruvate slightly increased the cell viability (Figure 2).

### 2.3. Evaluation of Double Combinations of Metabolic Inhibitors

**Figure 3:**
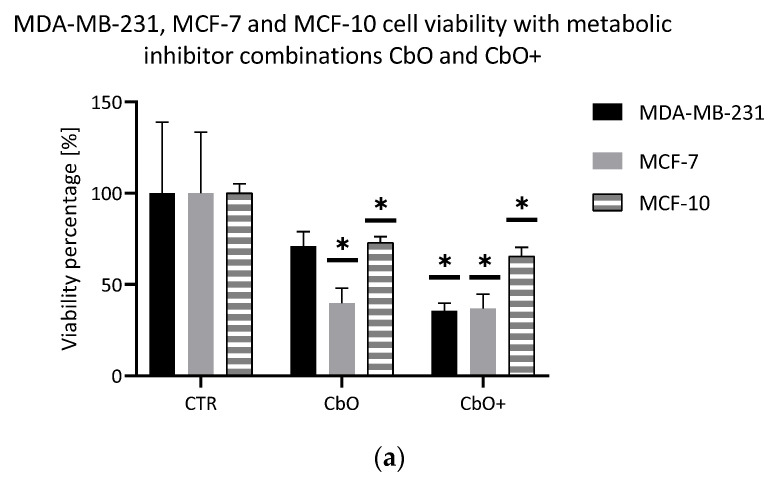

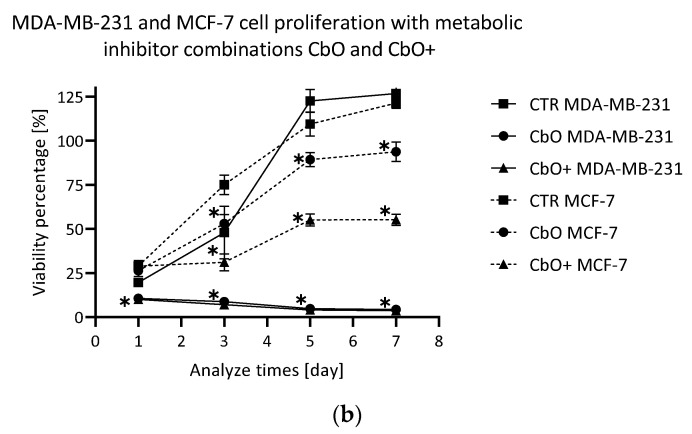
CbO double metabolic inhibitor combination evaluation. (**a**) Viability test (crystal violet staining) after 72 h. Black: MDA-MB-231, Grey: MCF-7 and Hatched: MCF-10; *n* = 6, average ± SD, significance: * *p* < 0.05. (**b**) Proliferation test (crystal violet staining) during 7 days of measuring at days 1, 3, 5 and 7. Full lines: MDA-MB-231 and Hatched lines: MCF-7; *n* = 6, average ± SD, significance: * *p* < 0.05. CTR: control condition, CbO: CB-839 50 nM and Oxamate 20 mM and CbO+: CB-839 1 µM and Oxamate 50 mM.

The first combination used in this study was the combination of CB-839 and Oxamate, called CbO. These metabolic inhibitors target both of the foremost metabolic pathways modified in cancer cells: the glutamine pathway and glucose and lactate pathways, respectively. Two concentration declinations were evaluated: a declination with both inhibitors used at their lowest or at their highest concentrations. This combination showed an excellent impact on the viability of both cell lines (Figure 3). The higher concentration declination decreased the viability to approximatively 35% in both cell lines. However, the MCF-7 cells seemed more sensitive to CbO than the MDA-MB-231 cells. Indeed, the effect on cell proliferation was different between the MDA-MB-231 and MCF-7 cells (Figure 3). In the first one, the proliferation curve was impacted and collapsed. In MCF-7 cells, the proliferation was significantly reduced, but the curve was not impacted as compared to the control curve.

The same combination was tested on the noncancer cell MCF-10. CbO and CbO+ (Figure 3) decreased viability in a significant way (72% and 65%, respectively).

Following these results, other double combinations were evaluated in the viability assay. Although some of them significantly reduced the cell viability, none did it better than CbO, which remains the most efficient double combination of metabolic inhibitors in this study (results not shown).

### 2.4. Evaluation of Triple Combinations of Metabolic Inhibitors

**Figure 4:**
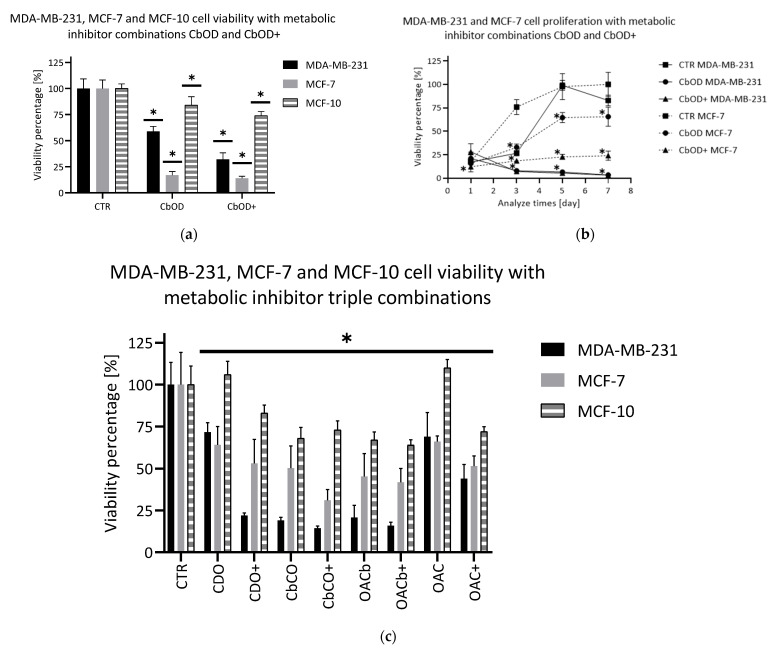
CbOD triple metabolic inhibitor combination evaluation. (**a**) Viability test (crystal violet staining) after 72 h. Black: MDA-MB-231, Grey: MCF-7 and Hatched: MCF-10. (**b**) Proliferation test (crystal violet staining) during 7 days of measuring at days 1, 3, 5 and 7. Full lines: MDA-MB-231 and Hatched lines: MCF-7. (**c**) Viability test (crystal violet staining) after 72 h. Black: MDA-MB-231, Grey: MCF-7 and Hatched: MCF-10. Conditions: CTR: control condition; CbOD: CB-839 50 nM, Oxamate 20 mM and D609 25 µM; CbOD+: CB-839 1 µM, Oxamate 50 mM and D609 75 µM; CDO: Chrysin 1 µM, D609 25 µM and Oxamate 20 mM; CDO+: Chrysin 5 µM, D609 75 µM and Oxamate 50 mM; CbCO: CB-839 50 nM, Chrysin 1 µM and Oxamate 20 mM; CbCO+: CB-839 1 µM, Chrysin 5 µM and Oxamate 50 mM; OACb: Oxamate 50 mM, AZD3965 5 nM and CB-839 50 nM; OACb+: Oxamate 50 mM, AZD3965 50 nM and CB-839 1 µM; OAC: Oxamate 20 mM, AZD3965 5 nM and Chrysin 1 µM and OAC+: Oxamate 50 mM, AZD3965 50 nM and Chrysin 5 µM; *n* = 6, average ± SD, significance: * *p* < 0.05.

The triple combination of CB-839, Oxamate and D609, so-called CbOD, was next evaluated. The metabolic inhibitors target the three foremost metabolic pathways modified in cancer cells: glutamine, glucose and lactate and the choline pathways, respectively. Two concentration declinations were evaluated: a declination with all the inhibitors used at their lowest and at their highest concentrations. As expected, this combination showed a better effect than the double-combination CbO. The viability was decreased to a level lower than 20% for the MCF-7 cell line and around 30% for the MDA-MB-231 cell line with the high concentration declination (Figure 4). Regarding proliferation, the impact was different depending on the cell lines (Figure 4). On the MDA-MB-231 cell line, the proliferation curve was totally collapsed in comparison to the control proliferation curve. On the MCF-7 cell line, the proliferation was significantly decreased compared to the control curve, but the same curve profile was observed.

As already mentioned in the case of the double combinations, other triple combinations were evaluated. Among these triple combinations, four of them showed a significant effect close to the one observed with CbOD on the cell viability: the combination of Chrysine, D609 and Oxamate, called CDO; the combination of CB-839, Chrysine and Oxamate, called CbCO; the combination of Oxamate, AZD3965 and CB-839, called OACb and the combination of Oxamate, AZD3965 and Chrysine, called OAC. However, some differences were noticed depending on the exposed cell line (Figure 4), the MDA-MB-231 cell line being more sensitive to the treatment. Regarding cell proliferation, the impact was also different on both cell lines. The proliferation of MCF-7 cells was significantly reduced with the inhibitor combinations compared to the control conditions, but the curve shape was not impacted. In the MDA-MB-231 cell line, the most efficient triple combinations showed a strong impact on the proliferation, and the curve collapsed in comparison to the control curve. For the two other combinations, the proliferation was significantly reduced, but the curve shape was unaffected (results not shown).

Like the inhibitors and double combinations, the triple combinations were tested on the MCF-10 line. The triple combinations CbOD and CbOD+ (Figure 4) showed a significant reduction of the number of viable cells (84% and 74% of the viability, respectively). In addition, other triple combinations showed a statistically significant impact on the MCF-10 viability, but the minimum threshold achieved only approximates 64% for OACb+ (Figure 4).

### 2.5. Potentiating Doxorubicin Efficacy with Metabolic Inhibitors

**Figure 5:**
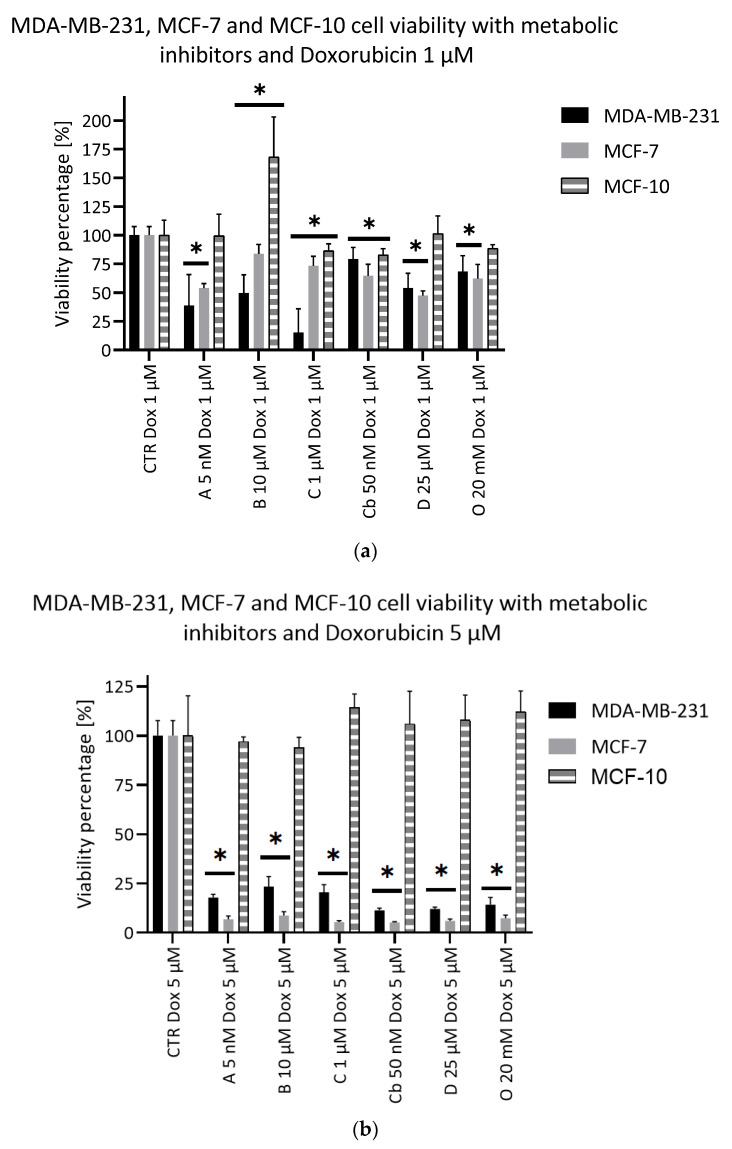
Potentiation of Doxorubicin (**a**) 1 µM and (**b**) 5 µM thanks to the metabolic inhibitors after 72 h (crystal violet staining). Black: MDA-MB-231, Grey: MCF-7 and Hatched: MCF-10. CTR: control condition. A: AZD3965, B: 3-bromopyruvate, C: Chrysin, Cb: CB-839, D: D609, O: Oxamate and Dox: Doxorubicin; *n* = 6, average ± SD, significance: * *p* < 0.05.

The potentiation of Doxorubicin by the selected metabolic inhibitors was evaluated thanks to the viability assay. Doxorubicin was used at 1 µM and 5 µM, and metabolic inhibitors were used at their lower concentrations. Four inhibitors used in combination with Doxorubicin at 1 µM were able to significantly increase the MDA-MB-231 cell mortality rate as compared to Doxorubicin alone: D609, 3-bromopyruvate, AZD3965 and Chrysine (Figure 5). In combination with Doxorubicin 5 µM, all the inhibitors displayed a significant additional effect on the cell viability (Figure 5). Regarding the MCF-7 cell line, AZD3965 and D609 only significantly impacted the cell viability when given with Doxorubicin 1 µM. For a Doxorubicin concentration of 5 µM, the results were the same as those observed with the MDA-MB-231 cell line. The Doxorubicin potentiation was also evaluated on the MCF-10 cells (Figure 5). At the lowest tested dose of Doxorubicin (1 µM), AZD3965, D609 and Oxamate had no significant effect on the cell viability compared to Doxorubicin alone. On the contrary, Chrysine and CB-839 significantly impacted the cell viability, although to a limited extent (86% and 82%, respectively, compared to Doxorubicin alone). Finally, 3-bromopyruvate significantly improved the MCF-10 cell viability. At the highest tested dose of the anticancer agent (5 µM), none of the inhibitors brought any additional effects to Doxorubicin.

### 2.6. Impact of Doxorubicin and Combinations of Metabolic Inhibitors on Cell Metabolism

**Figure 6:**
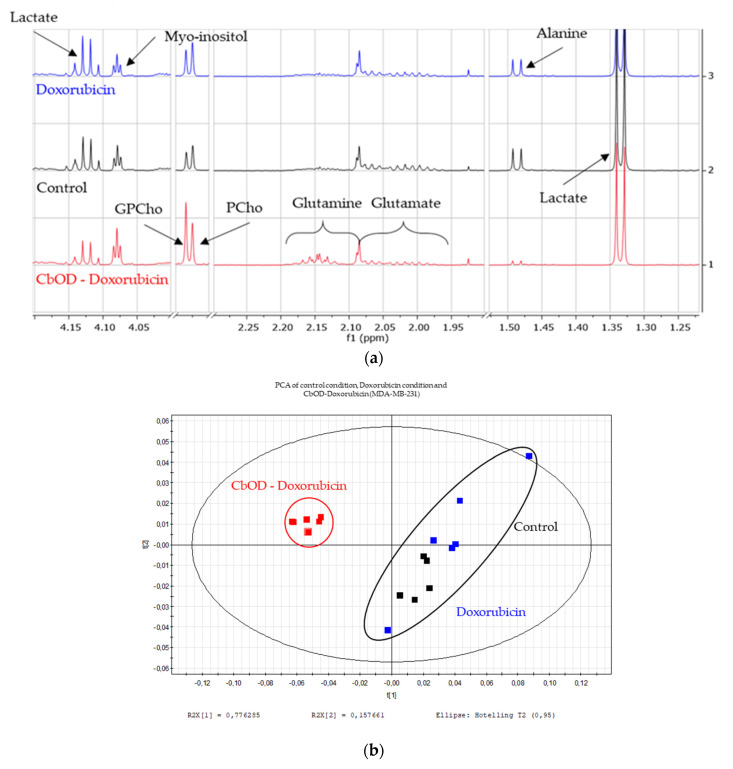

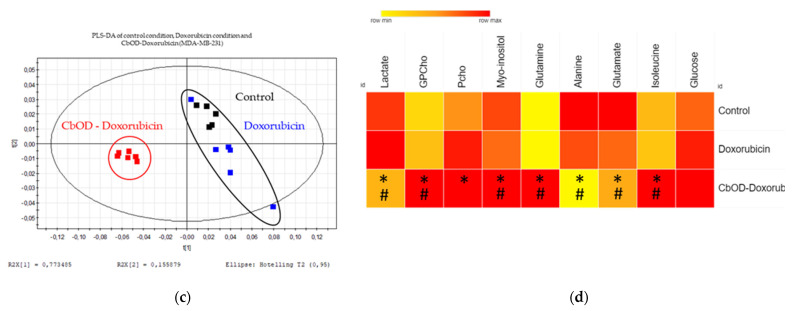
Impact on MDA-MB-231 metabolism. (**a**) The 600-MHz ^1^H-NMR spectra of the intracellular polar extract of cells treated with CbOD-Doxorubicin, with Doxorubicin and the control cells showing a metabolite variation with a VIP ≥ 1. (**b**) Scores plot (PCA) of the intracellular spectra of the samples from the control cells (black), Doxorubicin-treated cells (blue) and CbOD/Doxorubicin-treated cells (red) in 234 variables. (**c**) Scores plot (PLS-DA) of the intracellular spectra of samples from the control cells (black), Doxorubicin-treated cells (blue) and CbOD/Doxorubicin-treated cells (red) in 234 variables. (**d**) Heatmaps of the discriminant metabolites identified between the 3 conditions. *n*= 6 and significance: * *p* < 0.05 for CbOD-Doxorubicin in comparison to the control; # *p* < 0.05 for CbOD-Doxorubicin in comparison to Doxorubicin.

The ^1^H-NMR analysis of the MDA-MB-231 cell line shows the impact of the metabolic inhibitor combination CbOD with Doxorubicin and Doxorubicin alone on the metabolism (Figure 6). At this low concentration, Doxorubicin alone does not induce any metabolic variation in comparison with the control conditions. On the contrary, the Doxorubicin/CbOD combination shows some metabolic variations: a lactate and a glutamine concentration increase and a glutamate concentration decrease. These effects correspond to the expected effects for the Oxamate and CB-839 inhibitors. The concentration of PCho decreases in the CbOD–Doxorubicin condition in comparison to the control condition. On the other side, the GPCho concentration increases with the CbOD–Doxorubicin combination.

The multivariate analyses confirmed these observations. As shown in Figure 6b, the PLS-DA allows the separation of the three studied conditions (Q² = 0.934, R²X = 0.991; R²Y = 0.993, *p*-value from the ANOVA = 0.0012). This separation was already shown on the PCA. The CbOD–Doxorubicin group shifted from the other groups, suggesting a different metabolic impact as compared to nonexposed cells and cells exposed to Doxorubicin alone, which displayed similar metabolisms. The scores plot shows this separation, and the metabolites with a VIP ≥ 1 given by the loadings plot were studied in a heatmap. The values in the heatmap are obtained thanks to the peak picking tool in the software. This additional analysis allowed PCho and GPCho to be picked up separately (Spectrum sections 3.22 and 3.23, respectively). The results are presented in Figure 6c.

The observed effects are most likely due to the inhibitor combination, since the NMR spectra obtained from the cells treated with Doxorubicin alone did not show any variations as compared to the unexposed cells. The multivariate analyses confirmed this assumption, since no group separation was noticed according to the reiterated PLS-DA (results not shown).

**Figure 7:**
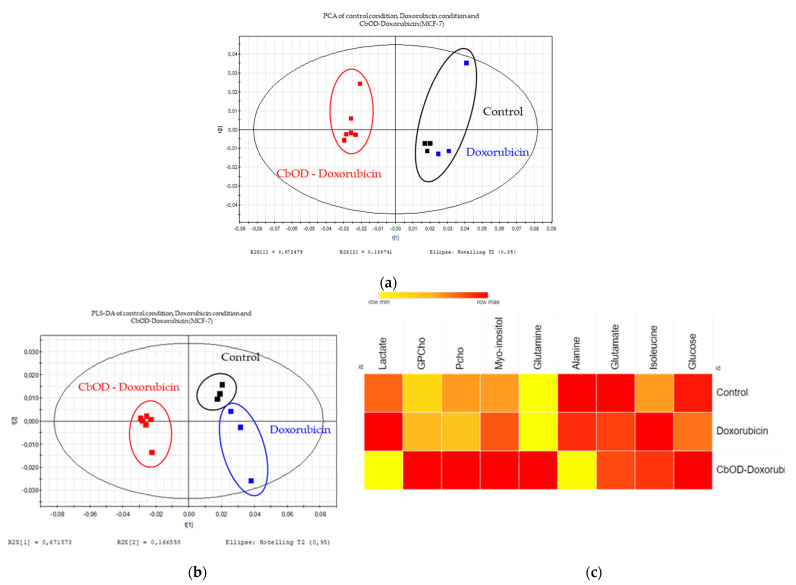
Impact on the MCF-7 metabolism. (**a**) Scores plot (PCA) of the intracellular spectra of the samples from the control cells (black), Doxorubicin-treated cells (blue) and CbOD/Doxorubicin-treated cells (red) in 234 variables. (**b**) Scores plot (PLS-DA) of the intracellular spectra of the samples from the control cells (black), Doxorubicin-treated cells (blue) and CbOD/Doxorubicin-treated cells (red) in 234 variables. (**c**) Heatmaps of the discriminant metabolites identified between the 3 conditions. *n* = 3 for Doxorubicin and the control conditions, and *n* = 6 for the CbOD-Doxorubicin condition.

A qualitative control of these multivariate analysis was carried out with the MCF-7 cell line. A PLS-DA with all the groups showed a good separation, but this separation was not significant (Q² = 0.954, R²X = 0.973, R²Y = 0.996, *p*-value from the ANOVA = 0.059) (Figure 7). Like MDA-MB-231, a separation was shown on the PCA, and the discriminant metabolites with a VIP ≥ 1 highlighted in the loadings plot were used to build a heatmap (value of peak picking) (Figure 7). These discriminant metabolites are essentially the same for both cell lines, except glutamate, which increases under the combination condition in comparison to the control condition. In addition, the PLS-DA comparing the unexposed and Doxorubicin-exposed cells control did not significantly separate the groups (results not shown).

## 3. Discussion

### 3.1. Highlighting Potential Targetable Metabolic Pathway

First, an ^1^H-NMR analysis was conducted on all the studied cell lines (MDA-MB-231, a triple-negative cell line and MCF-7, an estrogen and progesterone-dependent cell line) to pinpoint the metabolic pathways that could be pharmacologically targeted. A previous study was carried out in-house on the selected cell lines to highlight the metabolome differences between some cell lines of different cancers [28].

As expected from the cancer hallmarks, the glucose pathway, Warburg effect, glutamine pathway and choline pathway showed up [9,13,29,30]. Hence, some metabolic inhibitors were selected accordingly: Oxamate, AZD3965 and 3-bromopyruvate, to impact the glucose metabolism and Warburg effect with, respectively, an inhibition of lactate dehydrogenase, of the MCT transporter and of the hexokinase enzyme; D609 to target the choline metabolism with an inhibition of phosphatidylcholine-specific phospholipase C; CB-839 to inhibit the glutamine metabolism via an inhibition of glutaminase and, finally, Chrysine against HIF-1α, a transcription factor implicated in numerous cell processes, especially in the metabolisms.

### 3.2. Evaluation of the Efficacy of Metabolic Inhibitors and Their Ability to Potentiate Doxorubicin

Among the metabolic inhibitors used in this study, some of them are being evaluated in preclinical trials like AZD3965 [25] or in clinical trials like CB-839 [31,32].

AZD3965 was already evaluated on other cancers like gastric and lung cancers with convincing efficacy [33]. Some other studies also investigated the combination of this inhibitor with the currently used therapy, such as Doxorubicin, Rituximab, Phenformin and Metformin on various cancer types, including breast and lung cancers [25,33]. In the present study, AZD3965 failed to impact both the viability and the proliferation of MDA-MB-231 and MCF-7 cells. Nevertheless, this inhibitor induced a significant potentiation of the Doxorubicin effect, since both parameters were more affected by the combination as compared to the drug alone.

3-bromopyruvate has already been used on various cancers like melanoma, mesothelioma, glioblastoma and gastric, pancreatic and breast cancers [34]. This inhibitor significantly potentiated the effects of well-known drugs (Tamoxifen, Chloroquine, 5-fluorouracil and Doxorubicin) on the growth of different cancer types (breast and colon cancers and neuroblastoma) [26,34,35]. In our study, although the 3-bromopyruvate effect was limited in monotherapy, our findings confirm its potentiation ability when given in combination with Doxorubicin as compared to the drug alone in the studied cell lines.

Among the tested molecules, Chrysin successfully impacted the cell viability in monotherapy, yet with a limited impact on the proliferation. This finding is the opposite of the results from another study conducted on the MCF-7 cell line [36,37]. As for a combined effect with Doxorubicin, the couple Chrysin/Doxorubicin drastically decreased the cell viability to an extent comparable to the effect obtained on breast cancer when Chrysin was given in combination with Metformin [38].

CB-839 was previously studied by our group [28]. The lack of activity on the cell viability and proliferation described then is confirmed. In addition, the present study reveals that CB-839 does not impact cell proliferation either. However, in combination with Doxorubicin, CB-839 further decreases the cell viability as compared to Doxorubicin alone, yet only at a high dose of the anticancer agent. This result is relevant, because combinations of this glutaminase inhibitor and anticancer agents reached clinic trials in the context of the treatment of breast and kidney cancers [31,32].

Since phosphatidylcholine-specific phospholipase C, the D609 target, is more ex-pressed in the MDA-MB-231 cell line than in the MCF-7 cell line [39], we expected a better effect of D609 on the MDA-MB-231 cell viability. However, such an effect was not observed under our experimental conditions. This study is also the first to evaluate the combination of D609 with an anticancer agent. A PC-PLC inhibitor does potentiate the Doxorubicin effect.

Last, but not least, our evaluation of Oxamate is in complete contradiction with the results at the same dose of the literature. Indeed, the literature reports that Oxamate does not impact the MCF-7 cells but induces a decrease of the MDA-MB-231 cell viability in vitro and in vivo in graft mice [40]. In the present study, the opposite phenomenon was observed: the cell viability was decreased in MCF-7 cells but not in MDA-MB-231 cells. However, Oxamate significantly decreased MCF-7 proliferation and practically abolished the proliferation of the MDA-MB-231 cells. In combination with known anticancer agents, Oxamate showed a stronger effect on the cell viability when given in combination with Doxorubicin and Metformin on breast cancer [27]. Similar results were observed in combination with Doxorubicin on chondrosarcoma and in combination with Phenformin on various cancers [27,40,41]. In our study, Oxamate did potentiate Doxorubicin, yet at a high dose of the anticancer drug.

Inhibitors showed a low impact on MCF-10. MCF-10 is a noncancer breast cell line. We used this line as a control. Consequently, the use of these metabolic inhibitors could not impact healthy tissue in a patient breast with a potential administration of these molecules. The proliferation was not studied, because the most important aspect in healthy tissue to protect is to not death cells. An impact on the cell proliferation ability is less severe.

### 3.3. Evaluation of the Combinations of Metabolic Inhibitors

#### 3.3.1. CbO and CbOD Combinations

First, the combination of Oxamate (O) and CB-839 (Cb) demonstrated an excellent efficacy on the same studied cell lines in a previous study carried out in our laboratory [28]. The present study further evaluated this combination effect on cell proliferation. A strong antiproliferative effect was recorded, as evidenced by the collapse of the cell proliferation.

Then, the triple combination of Oxamate (O), CB-839 (Cb) and D609 (D) was investigated and showed a stronger effect as compared to the double CbO combination. This triple combination presents a good efficacy to reduce both cell line viabilities; yet, the proliferation is only impacted by the MDA-MB-231 cells. The MCF-7 cell proliferation is reduced due to an impact on the cell viability. This combination, like other triple combinations, was explored for the first time in this study.

#### 3.3.2. Other Triple Combinations

Double and triple combinations of the six inhibitors used in this study were tested on the MCF-7, MDA-MB-231 and MCF-10 cell lines. All the double combinations showed a decrease of the cell viability, with the best efficacy with CbO. Four triple combinations showed a good effect on the cell viability, some of them to the same extent as observed with CbOD.

#### 3.3.3. Conclusion about Metabolic Inhibitor Combinations

The MCF-7 cells are more sensitive to the tested inhibitors alone or in combination (CbO and CbOD) than the MDA-MB-231 cells, as demonstrated in the viability assay. However, other combinations have a slightly weaker effect on the MDA-MB-231 than MCF-7 cell viability and a stronger antiproliferative effect on the MDA-MB-231 than MCF-7 cells. Therefore, globally, metabolic inhibitor combinations are more efficient on MDA-MB-231 than MCF-7 cells—thus, on the more aggressive cell line. In addition, since all combinations that impacted the cell proliferation contained CB-839, one can assume that this inhibitor is responsible of the antiproliferative effect.

Like the metabolic inhibitor as monotherapy, inhibitor combinations significantly decrease the viability of MCF-10 but with a lower effect than on cancer cell lines. The conclusion is the same as the precedent point: healthy tissue in a breast patient could be impacted by potential combination therapy but in a lower way than the tumor.

### 3.4. Impact of Doxorubicin and Inhibitor Combinations on Metabolism

The Doxorubicin effect on the cell metabolism has already been studied on H9C2 cardio-myoblasts [24]. In this study, we focused on breast cancer cell lines. Since the MDA-MB-231 line is generally more sensible to inhibitor combinations and Doxorubicin than MCF-7, the foremost semiquantitative metabonomic analysis was carried out on this cell line, and a qualitative confirmation was performed on the second cell line.

Doxorubicin did not have any effect on the cell metabolism at the low tested dose, as suggested by the multivariate data analyses of the ^1^H-NMR spectra from the cellular extracts. In combination with CbOD, Doxorubicin had an impact on the metabolism. However, those observed effects were only due to the inhibitors. Mostly, decreases in the lactate and glutamate contents, as well as some increase in glutamine and fluctuations in the PCho-GPCho ratio, were observed and matched the cell responses expected for each inhibitor. By inhibiting lactate dehydrogenase A, Oxamate stops the conversion of pyruvate to lactate [40], which is lowered. CB-839 inhibits glutaminase and stops the transformation of glutamine into glutamate [9], resulting in a higher level of glutamine and a concomitant decrease of the glutamate content. In addition, since alanine is also produced from glutamine [9], the inhibition of the glutamine metabolism causes a reduction in alanine synthesis. Finally, PCho and GPCho are increased according to what is expected from the mode of action of D609. Furthermore, a variation in the ratio of both metabolites was observed. In cancer cells, PCho is more abundant than GPCho, but, in cells exposed to anticancer drugs, this ratio gets inverted [11]. As a matter of fact, this inverted ratio was noticed in our results with a PCho/GPCho ratio of 1.34 in the control cells and a ratio of 0.72 in the MDA-MB-231 cells exposed to a combination CbOD/Doxorubicin. The inversion was not observed in MCF-7 cells for which only a decrease of the ratio (1.4 for the control condition and 1.16 for the combination condition) was seen. However, since D609 targets the PC-PLC enzyme responsible for the conversion of PtdCho to PCho [42], a decrease in PCho was expected but not observed under our experimental conditions.

Myo-inositol is also involved in the metabolic signature of cells exposed to combined metabolic inhibitors. Myo-inositol is produced from glucose like lactate in BRAF-mutated cells (MDA-MB-231): lactate supplies ATP production, and myo-inositol is used to produce phosphoinositides, further used as a second messenger by the cells [28]. Oxamate inhibits the lactate production in favor of myo-inositol.

It is worth mentioning that the glutamate level was increased in MCF-7 cells treated with CbOD-Doxorubicin, while it decreased in the MDA-MB-231 cells as compared to the unexposed cells. Contrary to the MDA-MB-231 cells, MCF-7 cells produce glutamate from ammonia and α-ketoglutarate [43]. Therefore, we checked the resonances of α-ketoglutarate in the NMR spectra of the MCF-7 and MDA-MB-231 cell extracts. Unexpectedly, the AUC of the α-ketoglutarate peaks were increased in the MCF-7 cellular extracts, possibly due to the overactivation of the Krebs cycle attempting to overproduce α-ketoglutarate.

## 4. Materials and Methods

### 4.1. Cell lines and Culture

This study was carried out on the MDA-MB-231 (ATCC^®^ HTB-26TM) and MCF-7 (ATCC^®^ HTB-22TM) cell lines. Cells were grown in a 75-cm^2^ flask in DMEM High-Glucose medium (Thermo Fisher 1196, Merelbeke, Belgium) with 4 mM of Glutamine added (Thermo Fisher, 25030-024, lot 2300449), 10% of fetal bovine serum (Gibco, lot 42G8378K) and a mix of 100 U/mL of penicillin and 100 µg/mL of streptomycin (Thermo Fisher, 15140122). As a noncancer control cell line, the MCF-10 breast cell line was kindly supplied by the Bordet Institute (Brussels, Belgium). Cells were grown in MEBM medium (Lonza CC-3151, Basel, Switzerland) with some supplements like BPE, hEGF, Insulin, Hydrocortisone and GA-1000 (Lonza CC-4136, Basel, Switzerland) added and 10% of fetal bovine serum (Gibco, lot 42G8378K).

### 4.2. Inhibitors

Specific inhibitors targeting the metabolic pathways of interest were selected: Oxamate, a lactate dehydrogenase inhibitor (Sigma-Aldrich, St. Louis, MO, USA), Chrysin, a HIF-1α inhibitor (Sigma-Aldrich, St. Louis, MO, USA), 3-bromo-pyruvate, a hexokinase inhibitor (Sigma-Aldrich, St. Louis, MO, USA), D609, a phosphatidylcholine-specific phospholipase C inhibitor (Gentaur, Kampenhout, Belgium), AZD3965, a MCT transporter inhibitor (Gentaur, Kampenhout, Belgium) and CB-839, an inhibitor of glutaminase (Cayman Chemicals, Ann Arbor, MI, USA). All the inhibitors were solubilized in DMSO except Oxamate, which was solubilized in a culture medium. The low and high doses were defined for every inhibitor (Table 1) according to a literature review and the in-house assays. Inhibitors were used alone or in combination either with other inhibitors or with Doxorubicin. Doxorubicin was used at the doses of 1 and 5 µM, based on an in-house dose range study. For every combination, low- and high-dose declinations were performed.

### 4.3. ^1^H-NMR Sample Collection and Extraction

The cells were seeded in a 75-cm² flask at the rate of 10^6^ cells/flask for MDA-MB-231 (*n* = 6 for each condition) and for MCF-7 (*n* = 6 for the CbOD–Doxorubicin condition and *n* = 3 for the other conditions). The cells were grown during the proliferation time at 37 °C under 5% CO_2_ until reaching confluence. The medium was removed and replaced with fresh medium added with Doxorubicin or an inhibitor combination with Doxorubicin. The cells were grown again for the exposure time (72 h) under the same conditions as those mentioned above. After the exposure period, the extracellular medium was removed and centrifuged to discard dead cells. The supernatant was conserved at −20 °C. The cells were washed twice with cold DPBS and then quenched with cold methanol and collected using a scraper. They were quenched for a second time by diving the Falcon tube in liquid nitrogen and then keeping it at −80 °C. A water–methanol–-chloroform (1:1:1) extraction was carried out in order to separate the polar metabolites from the apolar compounds. The polar phase was dried using a speed vacuum. The dried samples were suspended in 700 µL of phosphate buffer (0.2-M Na_2_HPO_4_/0.04-M NaH_2_PO_4_, pH 7.4) in a H_2_O/D_2_O (80:20) mix. The samples were preserved at -80 °C before the analysis.

Before the ^1^H-NMR analysis, intracellular media were added with 1 mM of 3-trimethylsilyl propionic-2,2,3,3-d4 acid (TSP) in 100% deuterium oxide. The extracellular medium was centrifuged during 3 min at 13,000× *g*. The supernatant recovered from this last centrifugation was mixed to phosphate buffer. The samples were added with 1-mM TSP. All samples were analyzed by ^1^H-NMR spectroscopy at 600 MHz (NMR spectrometer Advance 600 Bruker). The NOESYPRESAT-1D pulse sequence with 256 scans was used.

### 4.4. Spectra Processing

Spectra obtained with the ^1^H-NMR analysis were processed with MestreLab Research 10.0.2 software (MestreLab Research, S.L, Santiago de Compostela, Spain). After the phases and base line corrections, all the spectra were calibrated to the TSP resonance that was arbitrarily fixed at 0.0 ppm with an intensity of 100. The spectra regions from 4.50 to 5.50 ppm and from 2.69 to 2.78 ppm were excluded, because they correspond to the highly fluctuating water and DMSO regions, respectively. The spectra were binned in 250 small subregions (or descriptors) by a 0.04-ppm step. The integrated binned data were exported in Excel, where a normalization to the total spectral area (AUC) was performed. Finally, the results were imported in Simca-P+ 12.0 (Umetrics, Umea, Sweden) for further multivariate data analyses.

### 4.5. Multivariate Data Analysis, Metabolic Signature, Heatmap

Using SimcaP+ 12.0.1 software, a Principal Component Analysis (PCA) and Partial Least Square Discriminant Analysis (PLS-DA) were performed on the dataset. A scores plot and a loadings plot were obtained to check any possible group separations among the samples and all discriminating descriptors (metabolites), respectively. Only those descriptors (and, thus, metabolites) with a VIP (Variable Importance in Projection) greater than or equal to 1 were considered and identified. This identification was carried out thanks to the Human Metabolome Database (HMDB).

In order to establish a metabolic signature for every tested condition (Doxorubicin and combination), a Peak Picking analysis was also performed. This is a critical step to check the success of the binning procedure, as well as the significance of the discriminant metabolites. Thus, the AUCs of all the descriptors with a VIP ≥ 1 were individually measured. The obtained numerical data were submitted to statistical analyses using R Studio software. This statistical analysis was carried out following the same procedure later described for viability and proliferation assays.

With these results of Peak Picking, a heatmap was built thanks to Morpheus online software (Morpheus, Broad Institute, Cambridge, MA, USA). The average was calculated for each group, and, for each condition, a ratio was calculated so that the higher average is equal to 1. The result table was loaded on Morpheus to generate a heatmap.

### 4.6. Viability Assay

To evaluate the cell viability, the cells were seeded in 96-well plates at the rate of 4000 cells/well for MDA-MB-231, 6000 cells/well for MCF-7 and 5000 cells/well for MCF-10 (*n* = 6 for each condition). The cells were grown during 3 days at 37 °C under 5% CO_2_. The medium was removed and replaced with fresh medium added with the inhibitor alone or in combination. The cells were grown again for 3 days under the same conditions. Then, the cells were washed twice with PBS. They were fixed in glutaraldehyde 4% and stained with crystal violet 1%. Finally, a triton solution allowed the permeabilization of the cell membrane to the dye. The absorbance was measured at 570 nm.

### 4.7. Proliferation Assay

To evaluate the cell proliferation, the cells were seeded in 96-well plates at the rate of 4000 cells/well for the MDA-MB-231 cell line and 6000 cells/well for the MCF-7 cell line (*n* = 6 for each condition and each time point). The cells were grown during 24 h at 37 °C under 5% CO_2_. The medium was removed and replaced with fresh medium added with an inhibitor alone or in combination. The cells were grown again according to the same timing as for the viability assay. The viability was measured at the first, third, fifth and seventh days. The same staining used in the viability assay was applied.

### 4.8. Statistical Method

First, a Shapiro–Wilk test was applied to the data to evaluate the population. If the population was not normal, a nonparametric hypothesis test was used. If the population was normal, a supplemental Bartlett test was performed. If the variances were heteroscedastic, a nonparametric hypothesis test was applied. If the variances were homoscedastic, a parametric hypothesis test was selected. As nonparametric hypothesis tests, a bivariate Wilcoxon test or a Kruskal–Wallis test were used. As for the parametric hypothesis test, an independent Student’s *t*-test and an ANOVA were performed.

## 5. General Conclusions

For a few years, the metabolic reprograming in cancer cells has been demonstrated and added to the list of the cancer hallmarks, as well as apoptosis evading, angiogenesis or growth signal independence. In fact, six metabolic hallmarks were defined. Some metabolic pathways are modified in cancerous cells like the glucose and lactate pathways with the Warburg effect, the glutamine pathway, the choline pathway, the amino acids pathway and the lipid pathway. Additionally, the expression of transcription factor HIF-1, which is largely involved in cell metabolism, is modified in cancer cells. These adaptations of cell metabolism support the tumoral initiation and maintenance. The metabolism indeed switches to promote survival, growth, proliferation and invasion. Due to such malignancies, cancer and, especially, breast cancer is still a public health issue.

In this study, we demonstrated that targeting cellular metabolism with specific enzyme inhibitors allows reducing the breast cancer cell viability. Although inhibitors given alone show a limited effect, double and triple combinations are more efficient. In particular, the double combination of CB-839 and Oxamate (CbO) and the triple combination of CB-839, Oxamate and D609 (CbOD) decrease the viability of MDA-MB-231 and MCF-7 cells, suggesting some clinical interest as future potential therapies. Some of the evaluated combinations also impacted the cell proliferation. This effect can be associated with CB-839, since other combinations without CB-839 only exerted cytotoxic effects on breast cancer cells. In addition, inhibitors and combinations induce a low impact on MCF-10 healthy cells.

We next successfully demonstrated the potentiation of the Doxorubicin anticancer effect by some metabolic inhibitors. These findings are encouraging with respect to the clinical objective of reducing the therapeutic dose of Doxorubicin and, consequently, the severity of cardiotoxicity in patients.

Finally, we carried out a metabonomic analysis that confirmed the expected cellular response to the CbOD combination: a decrease of the lactate production from pyruvate, a decrease of the glutamine metabolism and, consequently, a decrease of the glutamate production and an inverted PCho/GPCho ratio. If this combination should be developed as a potential future therapy, the treatment efficacy could be checked in patients by the ^1^H-NMR spectroscopy of patient’s biofluids and/or a tumor biopsy. Metabolic adaptations will be considered as biomarkers of the treatment efficacy.

## Figures and Tables

**Table 1:** Concentrations of the metabolic inhibitors.

Inhibitors	Low Concentration	High Concentration
Oxamate	20 mM	50 mM
Chrysin	1 µM	5 µM
AZD3965	5 nM	50 nM
D609	25 µM	75 µM
3-bromo-pyruvate	10 µM	50 µM
CB-839	50 nM	1 µM

## Data Availability

Publicly available datasets were analyzed in this study. This data can be found in MetaboLights database with accession number: MTBLS3338.
